# Identification of PGC-related ncRNAs and their relationship with the clinicopathological features of Gastric Cancer

**DOI:** 10.7150/jca.47787

**Published:** 2021-05-27

**Authors:** Han-xi Ding, Ye-feng Wu, Qian Xu, Yuan Yuan

**Affiliations:** 1 Tumor Etiology and Screening Department of Cancer Institute and General Surgery, the First Hospital of China Medical University, Shenyang 110001, China.; 2 Key Laboratory of Cancer Etiology and Prevention in Liaoning Provincial Education Department, the First Hospital of China Medical University, Shenyang 110001, China.; 3 Key Laboratory of GI Cancer Etiology and Prevention in Liaoning Province, the First Hospital of China Medical University, Shenyang 110001, China.

**Keywords:** CircRNA, MiRNA, Pepsinogen C, Gastric cancer, ceRNA network

## Abstract

Pepsinogen C (PGC) is considered to be the final product of mature differentiated gastric mucosa. The expression level of PGC in gastric mucosa is clearly decreased upon the development of gastric cancer (GC). However, the mechanism behind PGC's down-regulation remains unclear and needs to be clarified. This study aimed to identify PGC-related ncRNAs with the potential to be PGC post-transcriptional regulators and to further explore the association between these ncRNAs and the clinicopathological parameters of GC. Bioinformatic software was used to predict miRNAs binding specifically to PGC and circRNAs binding specifically to these candidate miRNAs. Dual-luciferase reporter assay was performed to validate the completely complementary pairing of PGC and PGC-related ncRNAs. qRT-PCR was applied to determine the expression levels of PGC and PGC-related ncRNAs in GC tissue. hsa-let-7c was predicted to bind to the PGC gene, which was confirmed by dual-luciferase reporter assay. hsa_circ_0001483 and hsa_circ_0001324 were identified to bind to hsa-let-7c by bioinformatic analysis and dual-luciferase reporter assay. In addition, the hsa_circ_0001483/hsa_circ_0001324 -hsa-let-7c-PGC axis was confirmed in tissue by qRT-PCR. The expression level of hsa_circ_0001483 was correlated with peritumoral inflammatory cell infiltration and lymphatic metastasis. hsa_circ_0001483, hsa_circ_0001324, and let-7c were newly identified and validated as PGC-related ncRNAs and showed associations with the clinicopathological features of GC. The hsa_circ_0001483/hsa_circ_0001324-hsa-let-7c-PGC axis in GC may account for the down-regulation of PGC in GC tissue.

## Introduction

Pepsinogen C (PGC), one of the mature forms of pepsinogen (PG), belongs to the aspartic protease family and is activated under acidic conditions 1-3. Under normal physiological conditions, PGC is mainly expressed in the whole stomach and is considered to be the final product of mature differentiated gastric mucosa 4. Multiple studies have demonstrated that PGC could play important roles in maintaining the normal morphology and physiological function of gastric epithelial cells 5, 6. According to our previous research, the expression level of PGC changes significantly in pathological condition, for example, PGC expression in gastric mucosa was found to decrease considerably during the transformation from superficial gastritis (SG) to atrophic gastritis (AG), and was even absent in gastric cancer (GC) 2. The low PGC level combined with the high expression of markers of a malignant phenotype such as MG7-Ag and MMP9 could be crucial molecular factors in the malignancy of gastric mucosa 6. In addition, protein chip arrays and SELDI-TOF MS suggested that PGC was clearly reduced in GC compared with that in normal gastric mucosa, which was confirmed by 2D electrophoresis and immunohistochemistry 7. Genome-wide association studies were conducted in the search for GC susceptibility loci and PGC was found to be a key gene in gastric epithelial differentiation. These findings indicated that PGC expression was negatively related to the occurrence and progression of GC 8. However, the mechanism behind the down-regulation of PGC remains unclear. Elucidation of how PGC gene expression is regulated could provide deeper insight into the pathogenesis of GC as well as aid in the identification of early diagnostic indicators and novel molecular targets for treating GC.

Non-coding RNAs (ncRNAs) play important roles in the post-transcriptional regulation of gene expression 9, 10. miRNAs are the most well-known ncRNAs, which are involved in the regulation of mRNA translation by binding to the 3′-untranslated region (3′-UTR), leading to the deregulation or suppression of mRNA transcripts 11, 12. circRNAs are another kind of ncRNAs that are widely present in eukaryotic cells, showing differences in expression among tissues types 13, 14. Many circRNAs contain miRNA binding sites, which can competitively bind to miRNA response elements (MREs), thus sequestering miRNAs from their target genes and further increasing the expression levels of target genes 13, 15. Owing to their function as miRNA sponges, circRNAs are closely associated with miRNAs and the relationships in circRNA-miRNA-mRNA networks have attracted substantial interest.

As an effective molecular marker in GC progression, exploration of the regulation of PGC expression by upstream ncRNAs may provide new insights into the genesis and development of GC. To date, a few PGC-related ncRNAs have been investigated. Liu et al. found that the serum expression level of lncRNAs adjacent to PGC could distinguish gastric cancer, atrophic gastritis and superficial gastritis 16. In addition, Lv et al. reported that 10 pairwise PGC-lncRNA single nucleotide polymorphisms (SNPs) were associated with gastric cancer risk, and might enhance the susceptibility to gastric cancer 17. Several other studies focused on the miRNAs targeting PGC and their relationship with gastric cancer. Some SNP or SNP-SNP interactions of these miRNAs showed significant correlations with the risk or prognosis of gastric cancer 18, 19. However, no evidence about the ncRNA network regulating PGC gene expression has yet been unearthed. In the present study, the ncRNAs involved in PGC regulation were predicted and verified, and the association between PGC-related ncRNAs and the clinicopathological characteristics of GC was further explored. This study showed help to clarify the regulatory mechanisms of PGC and provide clues for identifying ncRNA markers of GC diagnosis and prognosis.

## Materials and methods

### Target miRNAs and circRNAs for PGC by bioinformatic analysis

Two-stage bioinformatic analysis was performed to seek target miRNAs and circRNAs for PGC. First, the possible target miRNAs for PGC were predicted using eight software programs (i.e. MirTarBase, http://mirtarbase.mbc.nctu.edu.tw/php/index.php; DIANA-microT, http://diana.imis.athena-innovation.gr/DianaTools/index.php?r=microtv4/index; MicroRNA.org, http://www.microrna.org/microrna/getMirnaForm.do; miRDB, http://www.mirdb.org/; RNA22-HAS, https://cm.jefferson.edu/rna22/; TargetMiner, https://www.isical.ac.in/~bioinfo_miu/targetminer20.htm; TargetScan-vert, http://www.targetscan.org/vert_71/ and PicTar-vert, https://pictar.mdc-berlin.de/), and an miRNA was included when three or more software programs suggested that it might be a target ncRNA of the PGC gene 20-27. In the prediction of possible target circRNAs for miRNAs, miRanda prediction algorithms (http://www.microrna.org/microrna/home.do, Targetscan (http://www.targetscan.org/) and Starbase (http://starbase.sysu.edu.cn/) were employed to select the possible target circRNAs for candidate miRNAs 26, 28. TargetScan predicted miRNA targets based on the seed region. And miRanda was mainly based on the binding free energy of circRNAs and miRNAs, in which lower free energy indicated a stronger binding affinity between them.

### Binding interactions among circRNAs, miRNAs, and PGC by dual-luciferase reporter assay

Prior to the dual-luciferase reporter assay, a transfection experiment was conducted to narrow down the range of PGC-targeting miRNAs. First, the luciferase reporter gene system was used to construct a luciferase reporter plasmid (pmitGLO-PGC) for the PGC 3′-UTR region, and the mimics of candidate miRNAs from bioinformatic analysis were also constructed (Genoarray Technology, Soochow, China). Then, they were co-transfected into HeLa cells, and the intensity of reporter gene expression was quantitatively measured within 48 h. miRNAs that were positive in the above experiment were introduced into overexpression plasmids and co-transfected with the PGC 3′-UTR region reporter plasmid into AD293 cells for verification. miRNAs that were positive in both cell lines were regarded as candidate miRNAs for dual-luciferase validation experiments.

Dual-luciferase reporter assay was performed to verify the targeted binding among circRNAs, miRNAs, and PGC. Wild-type or mutant-type fragments in the PGC 3′-UTR associated with each candidate miRNA were designed, synthesized and inserted into GV306 vector (Genechem Co.,Ltd, Shanghai, China). Then, GV306 vectors and overexpression plasmids of candidate miRNAs were co-transfected into AD293 cells to determine the miRNA targets of the PGC gene. After 48 h, firefly and *Renilla* luciferase activities were measured, and the ratio of firefly luciferase intensity to *Renilla* luciferase intensity was calculated (E2910; Promega). SV40-firefly_luciferase-MCS vectors carrying wild-type or mutated hsa_circ_0001483, hsa_circ_0001324, hsa_circ_0001051, and hsa_circ_0001614 were co-transfected with hsa-let-7c overexpression plasmid and negative control (NC) plasmid into AD293 cells to identify circRNAs with the ability to bind to hsa-let-7c (Genechem Co.,Ltd, Shanghai, China) in a targeted manner. After 48 h, the luciferase activities of these reporters were determined using the dual-luciferase reporter assay system (E2910; Promega). The dual-luciferase reporter experiment was repeated three times in this round, and three times for each hole.

### Detection of circRNA, miRNA, and PGC expression *in vivo* and *in vitro*

#### Collection of clinical specimens and accessory information

Gastric cancer tissue and corresponding normal tissue (2 cm away from the tumor pathologically diagnosed by two pathologists) as well as accessory information were collected from 66 patients diagnosed with gastric cancer at The First Hospital of China Medical University (Shenyang; China) from November 2013 to September 2017. Enrolled subjects were pathologically diagnosed with gastric cancer by two qualified pathologists, in accordance with the World Health Organization (WHO) criteria. Patients with a history of other malignancies or undergoing preoperative radiotherapy or chemotherapy were excluded. Overall survival (OS) was followed up for 2 years. Informed consent was obtained from all participants before specimen collection. The study was approved by the ethics committee of the First Hospital of China Medical University.

#### Cell culture

The human GC cell lines AGS, HGC-27 and MKN-45 were purchased from the Institute of Basic Medical Sciences, Chinese Academy of Medical Sciences (Beijing; China). All cell lines were cultured in RPMI-1640 medium, with 10% fetal bovine serum (HyClone, USA). The cells were incubated at 37 °C in a humidified atmosphere containing 5% CO_2_.

#### RNA isolation and qRT-PCR

Total RNA was extracted using RNAiso Plus reagent, in accordance with the manufacturer's protocol (TaKaRa, Japan). cDNAs for circRNAs and mRNAs were synthesized by PrimeScript RT Master Mix with the following schedule: 37 °C for 15 min and 85 °C for 5 s (Perfect Real Time, cat#RR036A; TaKaRa, Japan). Relative expression levels of circRNAs and mRNAs were determined by TB Green Premix EX Taq II (TliRNaseH Plus, cat#RR820A; TaKaRa, Japan). For miRNAs, cDNA was synthesized by miRcute Plus miRNA First-strand cDNA Kit (cat#KR211; TIANGEN) and the relative expression level was measured by miRcute Plus miRNA qPCR Detection Kit (cat#FP411-02; TIANGEN). β-Actin and miR-16 were adopted as endogenous reference controls. All qRT-PCR curves were single peaks. The 2^-ΔCt^ method was used to calculate the relative expression level of cDNA. The primers for circRNAs are presented in Table S1.

### Statistical analysis

Statistical analysis was mainly performed by SPSSv18.0 (IBM, SPSS, and Chicago, IL, USA) and GraphPad Prism V5.0 software (GraphPad Software, USA). A *P*-value of < 0.05 was considered to be statistically significant. Student's t-test was used to evaluate the difference between two groups for normally distributed data, while the rank sum test was applied for data with a skewed distribution. The Kaplan-Meier method was employed to estimate the association of ncRNA expression with OS, and Cox regression was performed to identify the prognostic factors of OS.

## Results

### Identification of miRNAs targeting the PGC gene

**Figure 1:**
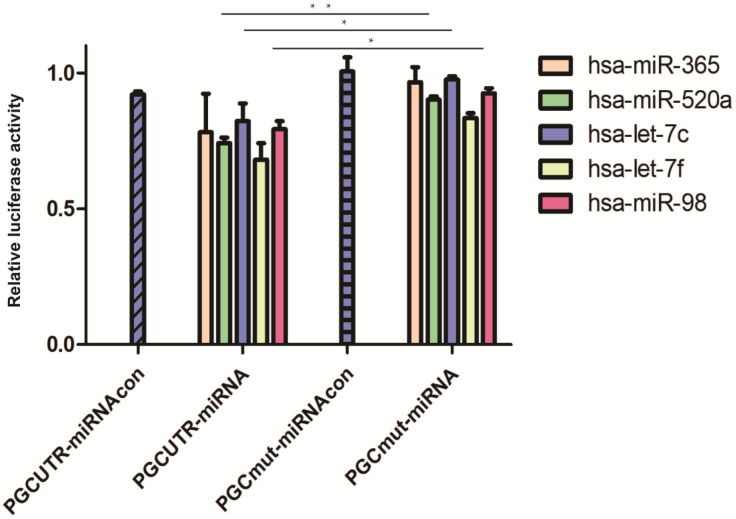
MiRNAs targeted to PGC in dual-luciferase reporter assay. Hsa-miR-520a, hsa-let-7c and hsa-miR-98 was significantly down-regulated in PGC wild-type group when compared with the PGC mutant-type (“**” means *p* < 0.010, “*” means *p* < 0.050).

A total of eight bioinformatic predictive software programs were utilized to seek miRNAs targeting the PGC gene; 39 possible target miRNAs were selected (Table S2). Then, the mimics of the candidate miRNAs were co-transfected with pmirGLO-PGC plasmid into the tool cells and nine miRNAs were screened out upon setting a threshold of PGC down-regulation to 90% (Table S3). The plasmids overexpressing these nine miRNAs (hsa-miR-662, hsa-miR-365, hsa-let-7f, hsa-miR-98, hsa-miR-525-5p, hsa-miR-520a-5p, hsa-let-7i, hsa-miR-126-5p, and hsa-let-7c) were synthesized and co-transfected with pmirGLO-PGC. Five miRNAs (hsa-miR-365, hsa-miR-520a, hsa-let-7f, hsa-let-7c, and hsa-miR-98) were identified upon setting a threshold of PGC down-regulation to below 85% (Table S4). Dual-luciferase reporter assay showed that hsa-miR-520a, hsa-let-7c, and hsa-miR-98 could impair the luciferase activity of PGC-UTR wild-type reporter, but not the mutant type (*P*=0.002, *P*=0.050, and *P*=0.020, respectively; Figure 1).

**Figure 2:**
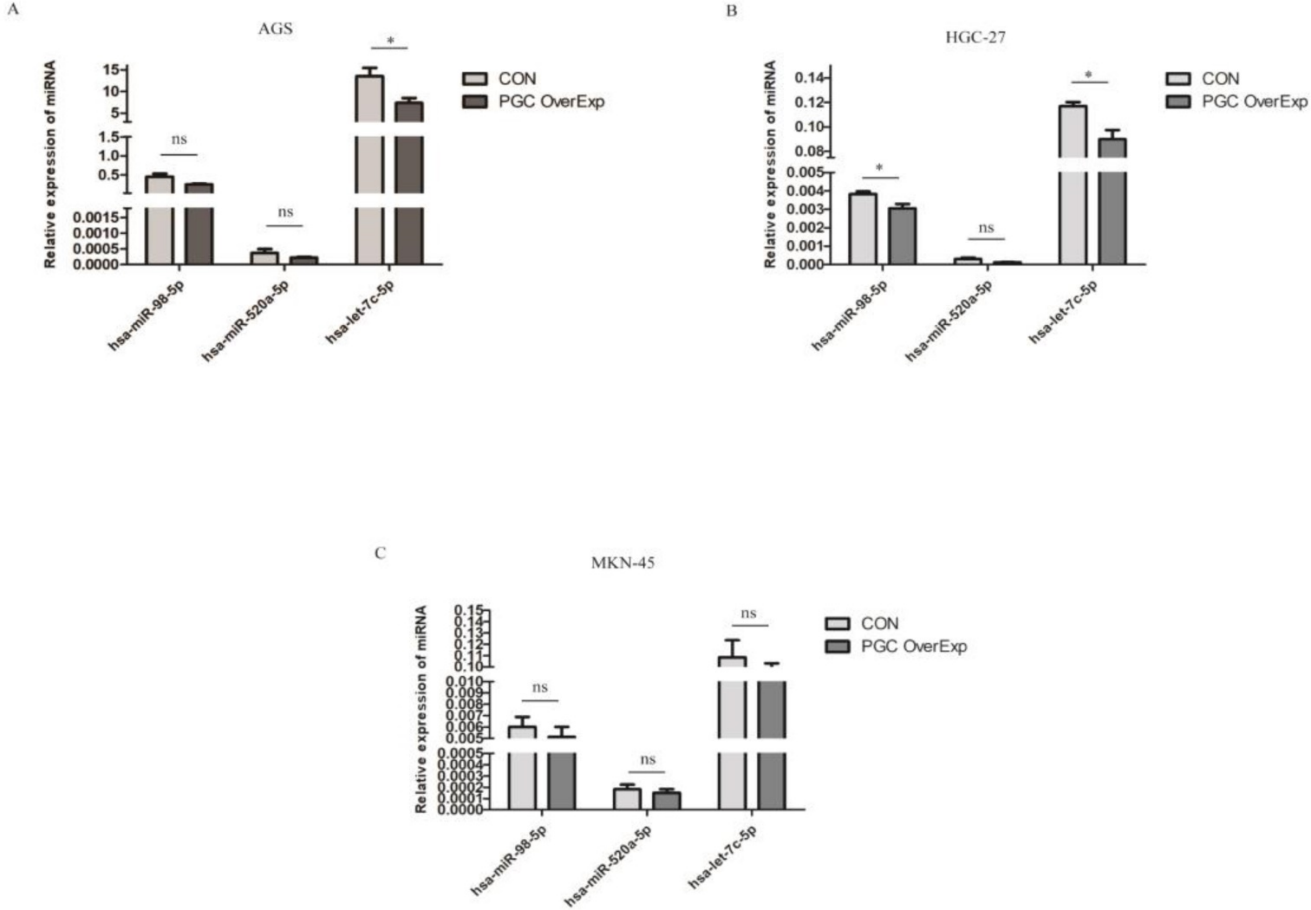
The expression level of hsa-let-7c when confected with PGC over-expression plasmid in AGS, HGC-27, and MKN-45 cells (“*” means *p* < 0.050; “ns” means no difference).

To identify the miRNAs differentially expressed after overexpressing the PGC gene, AGS, HGC-27, and MKN-45 cells lines were transfected individually with PGC-overexpression plasmid and NC plasmid. Compared with the level in the control group, hsa-let-7c was significantly down-regulated in the AGS cell line (*p*=0.050, Figure 2A), and hsa-let-7c and hsa-miR-98 were down-regulated in the HGC-27 cell line (*P*=0.011 and *P*=0.012, respectively; Figure 2B), while the expression of hsa-miR-520a, hsa-let-7c, and hsa-miR-98 was not significantly changed in the MKN-45 cell line (Figure 2C). From the results of cell transfection assays, we selected hsa-let-7c for further study. Fluorescent images of the cell transfection efficiency are provided in Supplementary Figure 1 (Figure S1).

### Identification of circRNAs targeted to PGC-related miRNAs

**Figure 3:**
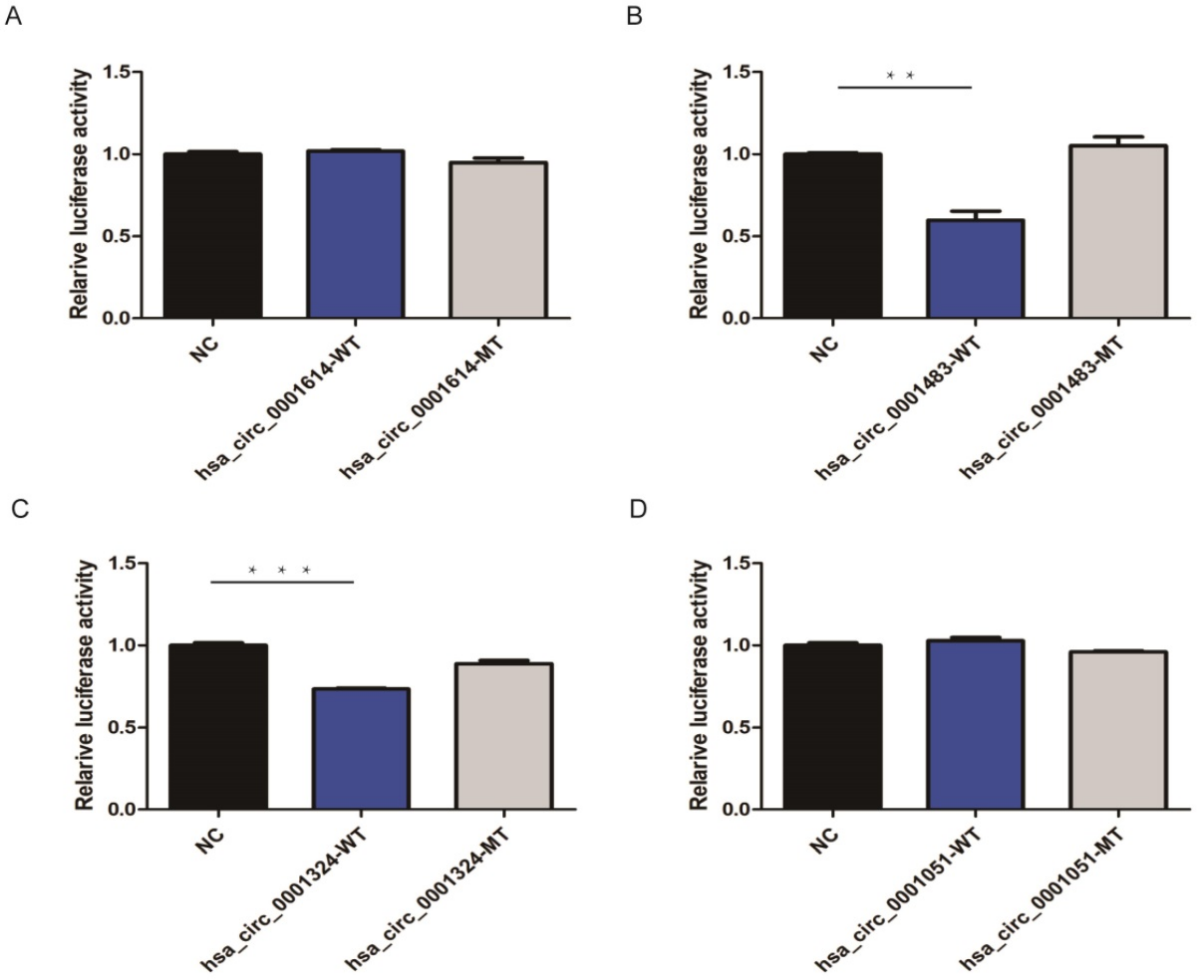
CircRNAs targeted to hsa-let-7c in dual-luciferase reporter assay. A, D. There was no significant reduction in luciferase reporter activity when compared hsa_circ_0001614-WT type and hsa_circ_0001051-WT type with control group. B, C. A significant reduction of luciferase reporter activity was observed in hsa_circ_0001483 and hsa_circ_0001324 wild-type reporter gene group than control group (“***” means *p* < 0.001, “**” means *p* <0.010).

Based on three bioinformatic software programs (TargetScan, miRanda, and starBase), we finally obtained 12 candidate circRNAs with targeted binding sites of hsa-let-7c for qRT-PCR validation. The expression of four of these circRNAs (hsa_circ_0012126, hsa_circ_0000365, hsa_circ_0000149, and hsa_circ_0002557) was too low in tissue to be detected by qRT-PCR, while the other seven circRNAs (hsa_circ_0001483, hsa_circ_0001610, hsa_circ_0001614, hsa_circ_0001685, hsa_circ_0000504, hsa_circ_0001324, and hsa_circ_0001051; Table 1) showed significant differences between 30 paired GC and normal tissues. Then, four circRNAs (hsa_circ_0001614, hsa_circ_0001483, hsa_circ_0001324, and hsa_circ_0001051) with the lowest* P*-values were chosen for subsequent dual-luciferase reporter assay to validate the targeted binding relationship with hsa-let-7c. Based on the predicted binding sites from RNAhybrid, the wild type or mutant type of the four circRNA fragments was constructed, inserted downstream of the reporter gene, and co-transfected with hsa-let-7c overexpression plasmid into 293T cells. The luciferase activity was significantly reduced in hsa_circ_0001483 and hsa_circ_0001324 wild type reporter gene groups (*p*=0.002 and *p* < 0.001, respectively; Figure 3).

### PGC-related ncRNA expression level and clinicopathological features of GC

**Figure 4:**
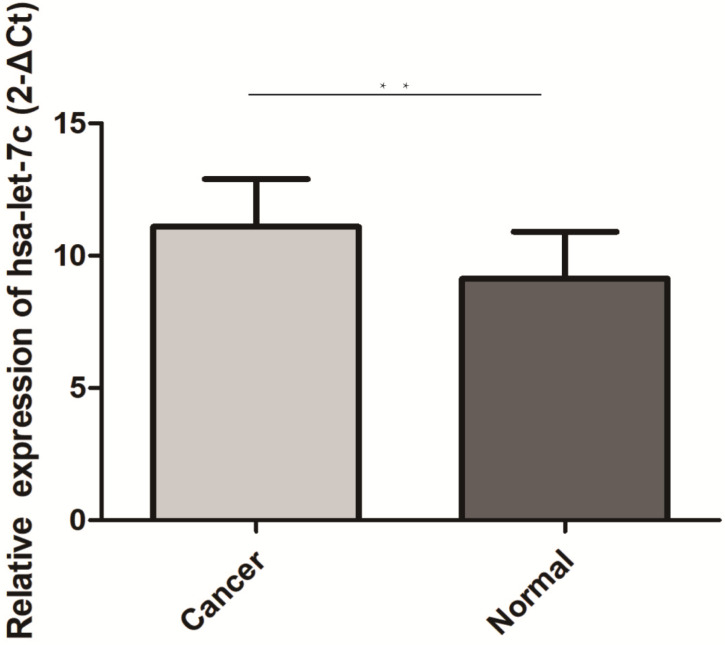
Hsa-let-7c was up-regulated in 30 pairs GC tissues (“**” means *p* < 0.010).

**Figure 5:**
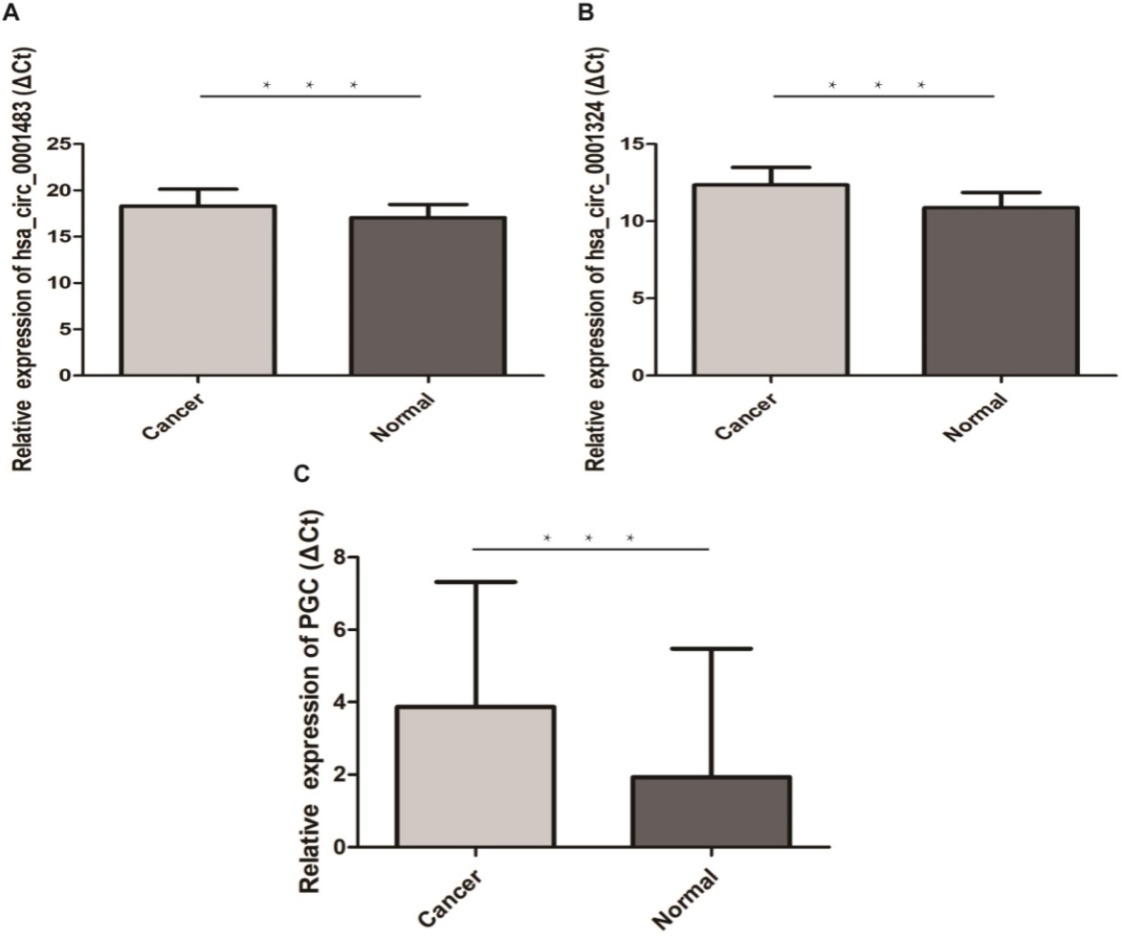
Hsa_circ_0001483, hsa_circ_0001324 and PGC was down-regulated in 66 pairs GC tissues (“***” means *p* < 0.001). The higher ΔCt value indicates a lower expression.

The qRT-PCR results of 30 paired GC and normal tissues suggested that hsa-let-7c was up-regulated in GC tissue (*p*=0.003; Figure 4). The expression levels of hsa_circ_0001483, hsa_circ_0001324, and PGC in 66 paired GC and distant normal tissues were also determined by qRT-PCR. As shown in Figure 5, both the two circRNAs and PGC were down-regulated in GC (*p* < 0.001). Moreover, a positive correlation was found between hsa_circ_0001324 and PGC in GC and normal tissues (*p* < 0.001, r=0.480 and *p* < 0.001, r=0.456, respectively; Table 2). In normal tissue, hsa_circ_0001483 was positively correlated with PGC expression (*p*=0.009, r=0.317; Table 2). However, no correlation of hsa-let-7c expression level with PGC, hsa_circ_000483, or hsa_circ_0001324 was observed.

In view of the diagnostic and prognostic roles of ncRNAs in GC, we also explored the clinical value of PGC-related ncRNAs. It was suggested that the expression level of hsa_circ_0001483 was negatively corrected with lymphatic metastasis (*p*=0.044, r=-0.249; Table S5). Additionally, hsa_circ_0001483 was highly expressed in the group with more severe pericarcinoma inflammatory cell infiltration and lymphatic metastasis (*p*=0.02 and *p*=0.044, respectively; Table 3). Besides, hsa_circ_0001324 was negatively correlated with patients' age (p=0.004, r= -0.350; Table S5). The expression level of hsa_circ_0001324 in the group ≥60 years old was lower than that in the group <60 years old (*p*=0.005; Table 3). However, the expression level of hsa-let-7c was not significantly correlated with the clinicopathological features (Table 3). We further analyzed whether the dysregulation of PGC-related ncRNAs could predict the prognosis of GC patients, while no significant association was observed between these ncRNAs and overall survival of GC (Tables S6 and S7).

We also performed independent external validation with TCGA database. The results suggested that PGC was negatively correlated with hsa-let-7c in pathological stages I and II (*P*=0.0274, r^2^=-0.316; *P*=0.0182, r^2^=-0.275, respectively).

## Discussion

PGC is a key gene in the process of epithelial differentiation of the stomach, and the regulatory mechanism involved in the significant down-regulation of PGC expression in GC requires in-depth investigation. In the present study, miRNAs with targeted binding sites of the PGC gene and circRNAs targeting these miRNAs were predicted. Dual-luciferase reporter experiments were performed to confirm the targeted binding relationship among PGC, miRNAs, and circRNAs. The expression patterns of PGC and PGC-related ncRNAs in GC tissue were also explored by qRT-PCR. Finally, let-7c, hsa_circ_0001483, and hsa_circ_0001324 were identified as PGC-related ncRNAs in GC, and their association with the clinicopathological characteristics of GC was also revealed.

Dysregulation of ncRNAs has been recognized as a novel molecular signature in cancer initiation and development. As a type of ncRNAs, miRNAs play an important role in down-regulating the transcription of target mRNAs. The relationship of miRNAs with GC has been demonstrated in previous reports 29. For example, miR-92a-1-5p was shown to increase CDX2 by targeting FOXD1 and to mediate gastric intestinal metaplasia 30. miR-143-3p may inhibit GC growth and confer greater sensitivity sensitive to cisplatin by targeting BRD2 31. Our study focused on the ncRNAs down-regulating PGC in GC tissue. Dual-luciferase reporter assay confirmed the findings of three miRNAs predicted by software to bind to PGC (hsa-miR-520a, hsa-let-7c, and hsa-miR-98). Accordingly, hsa-miR-520 was indicated to regulate the proliferation and migration of lung cancer and also modulate the progression of renal carcinoma. However, there was a lack of evidence that this miRNA is related to GC 32, 33. miR-98 could serve as a biomarker for prostate cancer diagnosis. It might be up-regulated in GC and thus involved in GC progression 34, 35. The Let-7 miRNA family plays a central role in regulating the relationship between miRNAs and mRNAs in GC, and hsa-let-7c was shown to be associated with the clinicopathological parameters of GC 36. Here, the above three miRNAs were first reported to be involved in PGC tissue expression. Our preliminary research showed that the serum hsa-let-7c expression level was inversely related to PGC 37. These PGC-related miRNAs may be involved in GC development by regulating PGC expression, although the specific mechanisms involved remain to be clarified. Nonetheless, they are potential biomarkers for GC.

circRNAs are another type of ncRNAs with unclear roles in cancer. They have been suggested to function as miRNA sponges in various cancers and are potential novel biomarkers 38-40. Many studies have reported that circRNAs exert regulatory roles in GC. Rong et al found that circHECTD1 activated β-catenin/c-Myc signaling by the adsorption of miR-1256, facilitated glutaminolysis, and thus promoted GC progression 41. In addition, circAKT3 was found to enhance cisplatin resistance in GC by suppressing hsa-miR-198 to up-regulate PIK3R1 42. In this study, the target circRNAs for hsa-let-7c were predicted by bioinformatic methods and 12 candidates were validated by qRT-PCR in 30 pairs of GC tissues. We selected four down-regulated circRNAs (hsa_circ_0001483, hsa_circ_0001324, hsa_circ-0001614, and hsa_circ_0001051) (*p*< 0.001) for dual-luciferase reporter assay with hsa-let-7c. The results showed that hsa_circ_0001483 and hsa_circ_0001324 could combine with hsa-let-7c. Based on dual-luciferase reporter assay, the PGC-specific ceRNA network was constructed, including hsa_circ_0001483/hsa_circ_0001324, hsa-let-7c, and PGC in GC. Consequently, hsa_circ_0001483 and hsa_circ_0001324 might down-regulate PGC expression by competitively binding to hsa-let-7c, and the two novel circRNAs could also serve as biomarkers for GC diagnosis.

The expression levels of hsa-let-7c, hsa_circ_0001483, hsa_circ_0001324, and PGC in GC tissues and distant normal tissues were further investigated by qRT-PCR. It was found that hsa_circ_0001483 and hsa_circ_0001324 were significantly down-regulated in GC tissue and had collinearity with PGC expression, indicating that their low expression in GC was consistent with the low expression of PGC. Moreover, hsa-let-7c was significantly overexpressed in GC tissues. These findings suggested possible endogenous interactions among these ncRNAs and the PGC gene. The abnormally low expression of hsa_circ_0001483 and hsa_circ_0001324 with high expression of hsa-let-7c in GC tissue makes more hsa-let-7c binds to the PGC-UTR region, leading to the down regulated expression of PGC. Furthermore, the expression level of hsa_circ_0001483 was associated with pericarcinoma inflammatory cell infiltration and lymphatic metastasis. Immune cell infiltration and metastasis are key events worthy of consideration when evaluating GC prognosis and therapeutic strategy 43-45. The dysregulation of hsa_circ_0001483 may influence immune response, chemosensitivity-related features, and the metastasis of GC patients, making it a potential biomarker for GC prognosis. In addition, higher expression of hsa_circ_0001324 and hsa_circ_0001483 was suggestive of better OS (*p*=0.070 and *p*=0.081, respectively). Considering a previous report describing that a low PGC expression level was correlated with shorter OS of GC patients 46, we believed that the low expression of hsa_circ_0001483 and hsa_circ_0001324 attenuated their capacity to absorb hsa-let-7c, thus down-regulating PGC expression, which may affect the prognosis of GC patients.

Some limitations of this study should be acknowledged. First, no complete analysis of the up- and down-stream targets involved in the related mechanisms was performed in this study, which could be focus of future research. In addition, our findings also require more independent validation by an external cohort, which warrant future investigation.

In conclusion, the PGC-related ncRNAs and their association with the clinicopathological parameters of GC were reported here for the first time. hsa_circ_0001483, hsa_circ_0001324, and let-7c were newly identified and validated as PGC-related ncRNAs, and they showed various associations with the clinicopathological features of GC. The hsa_circ_0001483/hsa_circ_0001324-hsa-let-7c-PGC axis might account for PGC down-regulation in GC tissue. Identification of the underlying molecular mechanisms underlying GC is of great significance for detecting therapeutic targets for management strategies. More importantly, further exploration of the biological functions of PGC, hsa-let-7c, as well as hsa_circ_0001324 and hsa_circ_0001483 could deepen our understanding of the pathogenesis of GC and also improve the diagnosis and treatment of this disease.

## Supplementary Material

Supplementary figure and tables.

## Figures and Tables

**Table 1:** Expression of 8 circRNAs in 30 pairs of gastric cancer and non-cancer tissues

Variable	CON		GC		
	N	ΔCt P_50_(P_25_, P_75_)	N	ΔCt P_50_(P_25_, P_75_)	*P*
hsa_circ_0001483	30	8.98 (8.18, 10.58)	30	12.11 (10.97, 13.31)	**0.001**
hsa_circ_0001610	30	5.45 (4.80, 6.06)	30	6.22 (5.44, 7.39)	**0.035**
hsa_circ_0001614	30	5.73 (5.14, 8.97)	30	7.93 (6.61, 8.97)	**<0.001**
hsa_circ_0001685	30	7.66 (7.08, 8.57)	30	9.54 (8.57, 10.71)	**0.002**
hsa_circ_0000504	30	7.43 (6.49, 8.12)	30	8.58 (7.65, 10.19)	**0.012**
hsa_circ_0001324	30	4.78 (3.76, 5.86)	30	7.37 (5.97, 8.52)	**<0.001**
hsa_circ_0001051	30	6.16 (5.13, 6.53)	30	8.01 (6.49, 8.83)	**0.001**
hsa_circ_0001355	30	3.63 (2.64, 4.28)	30	4.51 (3.11, 5.73)	0.250

Note: This table showed the expression differences of the eight circRNAs between gastric cancer and normal tissues. The higher ΔCt value indicates the lower expression. CON, control; GC, gastric cancer.

**Table 2:** Correlation among hsa-let-7c, hsa_circ_1324, hsa_circ_1483 and PGC

	CON		GC	
	r	*P*	r	*P*
hsa-let-7c and hsa_circ_1483	0.155	0.415	-0.253	0.177
hsa-let-7c and hsa_circ_1324	0.124	0.514	0.000	0.999
hsa-let-7c and PGC	0.057	0.766	-0.027	0.888
hsa_circ_1483 and PGC	**0.317**	**0.009**	0.202	0.104
hsa_circ_1324 and PGC	**0.456**	**<0.001**	**0.480**	**<0.001**

Note: The correlation analysis was performed by Spearman's correlation coefficient for the skewed distribution data. CON, control; GC, gastric cancer; PGC, pepsinogen C; r, Spearman's correlation coefficient.

**Table 3:** Relationship between the expression level of hsa-let-7c, hsa_circ_0001324 and hsa_circ_0001483 in gastric cancer patients and clinicopathological factors

Variability	Patient Number	hsa_circ_0001324(ΔCt ) p50(p25, p75)	*P*(2^ -ΔCt^)	Patient Number	hsa_circ_0001483(ΔCt ) p50(p25, p75)	*P*(2^ -ΔCt^)	Patient Number	hsa-let-7c(2 ^-ΔCt^) p50(p25, p75)	*P*(2 ^-ΔCt^)
**Gender**			0.910			0.189			0.901
Male	42	12.36 (11.41, 13.77)		42	18.20 (17.40, 19.68)		16	3.76 (0.98, 10.39)	
**Female**	24	12.41 (11.15, 13.37)		24	19.11 (17.78, 20.61)		14	2.50 (1.21, 28.84)	
Age			0.005			0.586			0.917
≥60	38	12.65 (11.81, 14.10)		38	18.93 (17.51, 20.37)		13	2.35 (1.11, 14.06)	
<60	28	11.40 (10.77, 12.82)		28	18.30 (17.44, 20.00)		17	2.97 (1.29, 16.53)	
**Smoking**			0.748			0.271			0.464
Ever Smoker	16	12.30 (11.67, 14.19)		16	19.65 (17.59, 20.81)		4	1.52 (0.46, 36.32)	
Never Smoker	50	12.43 (11.26, 13.26)		50	18.27 (17.44, 19.70)		26	2.97 (1.21, 12.27)	
**Drinking**			0.758			0.433			0.454
Ever Drinker	9	11.72 (11.68, 14.28)		9	19.67 (17.07, 20.93)		2	NA	
Never Drinker	57	12.44 (11.26, 13.39)		57	18.27 (17.50, 19.92)		28	2.97 (1.16, 15.84)	
**Family History**			0.866			0.449			0.707
Yes	15	12.52 (11.22, 14.05)		15	18.59 (18.24, 20.47)		8	2.33 (1.46, 4.62)	
No	51	12.22 (11.28, 13.34)		51	18.15 (17.44, 20.07)		22	3.31 (0.96, 26.33)	
**Location**			0.767			0.418			0.891
Body	20	12.28 (11.29, 13.41)		20	18.09 (16.94, 19.66)		13	2.03 (1.28, 7.62)	
Angle	11	11.84 (11.11, 12.64)		11	19.30 (18.47, 21.65)		3	NA	
Antrum	23	12.60 (11.24, 13.84)		23	18.26 (17.78, 19.67)		10	2.66 (1.40, 44.06)	
Entire	11	12.03 (11.31, 14.05)		11	19.59 (17.00, 20.61)		3	NA	
**Macroscopic Type**			0.856			0.718			0.565
Protruded Type	3	NA		3	NA		2	NA	
Ulcerative Type	13	12.44 (11.75, 12.86)		13	18.60 (17.17, 20.06)		6	2.15 (0.88, 16.99)	
Ulcerative Infiltrative Type	32	12.37 (10.72, 13.30)		32	18.53 (17.88, 20.22)		13	5.17 (1.57, 16.53)	
Diffuse Infiltrative Type	18	11.87 (11.27, 14.15)		18	18.30 (16.77, 19.77)		9	2.97 (1.18, 32.08)	
**Histological Type**			0.744			0.287			0.499
Papillary Adenocarcinoma (I)	0	NA		0	NA		0	NA	
Well Differentiated Type (II)	1	NA		1	NA		1	NA	
Moderately Differentiated Type (III)	8	12.39 (11.84, 12.90)		8	18.07 (17.53, 20.10)		2	NA	
Poorly Differentiated Type (IV)	39	12.38 (11.24, 14.09)		32	18.33 (17.42, 19.77)		15	1.69 (1.23, 8.51)	
Mucinous Adenocarcinoma (V)	6	12.73 (10.91, 13.30)		5	20.04 (18.57, 20.52)		4	2.50 (0.92, 8.34)	
Signet-ring Cell Cinoma (VI)	10	11.43 (10.88, 13.07)		9	17.87 (16.85, 19.18)		6	26.63 (3.37, 76.49)	
**Lauren Classification**			0.757			0.915			0.577
Intestinal	9	12.33 (11.84, 12.77)		9	18.15 (17.62, 19.84)		3	NA	
Diffuse	55	12.22 (11.22, 13.84)		55	18.47 (17.42, 20.07)		25	2.97 (1.28, 13.88)	
**TNM stage**			0.473			0.160			0.422
I	7	12.01 (10.71, 12.64)		7	19.30 (18.26, 22.69)		4	4.11 (0.78, 9.54)	
II	23	12.86 (11.11, 14.35)		23	18.15 (17.34, 20.37)		9	10.13 (1.34, 55.27)	
III	36	12.22 (11.34, 13.30)		36	18.30 (17.44, 19.70)		17	2.03 (1.18, 6.84)	
IV	0	NA		0	NA		0	NA	
			0.606			0.575			0.315
I+II	30	12.54 (11.07, 13.95)		30	18.54 (17.51, 21.06)		13	6.73 (1.24, 29.72)	
III+IV	36	12.22 (11.34, 13.30)		36	18.30 (17.44, 19.70)		17	2.03 (1.18, 6.84)	
**Peritumoral Inflammatory Cells**			0.685			0.03			0.159
+	18	11.97 (11.29, 12.89)		18	17.80 (16.85, 18.68)		9	8.51 (2.66, 42.26)	
++	26	12.41 (11.02, 13.42)		26	19.30 (18.15, 20.62)	0.02	11	1.69 (0.99, 5.17)	
+++	21	12.60 (11.42, 14.15)		21	18.32 (17.40, 20.43)		9	1.44 (1.00, 10.30)	
**Lymphovascular invasion**			0.931			0.984			0.485
+	42	12.37 (11.26, 13.77)		42	18.33 (17.43, 20.15)		18	2.19 (1.10, 8.91)	
-	24	12.36 (11.35, 13.29)		24	18.43 (17.48, 20.20)		12	4.85 (1.36, 33.11)	
**Ganglion invasion**			0.352			0.375			0.518
+	53	12.09 (11.23, 13.74)		53	18.32 (17.37, 20.38)		23	2.97 (1.34, 22.94)	
-	12	12.58 (12.09, 13.51)		12	18.53 (18.06, 19.59)		6	4.11 (0.79, 10.22)	
**Lymphatic metastasis**			0.332			0.044			0.944
+	45	12.22 (11.25, 13.39)		45	18.27 (17.42, 19.67)		22	2.19 (1.21, 12.00)	
-	21	12.64 (11.66, 13.76)		21	19.30 (17.87, 22.14)		8	4.85 (0.78, 19.83)	
**Depth of invasion**			0.583			0.264			0.937
Mucous Layer (pT1)	2	NA		2	NA		1	NA	
Submucosal Layer (pT2)	5	11.48 (10.34, 12.68)		5	18.03 (17.06, 18.10)		3	NA	
Muscular Layer (pT3)	7	12.52 (11.11, 12.86)		7	18.88 (18.47, 22.69)		3	NA	
Subserosa Layer (pT4)	1	NA		1	NA		0	NA	
Serosal Layer or Invasion Adjacent Organs(pT5)	51	12.52 (11.28, 14.09)		43	18.32 (17.40, 20.37)		23	2.35 (1.23, 22.94)	
			0.340			0.263			0.845
pT1+pT2	7	1201 (10.37, 12.44)		7	18.04 (17.45, 18.26)		4	2.23 (1.02, 5.79)	
pT3+pT4	8	12.18 (11.19, 12.81)		8	19.09 (18.50, 21.86)		3	NA	
pT5	51	12.52 (11.28, 14.09)		51	18.32 (17.40, 20.37)		23	2.34 (1.23, 22.94)	
**Growth Pattern**			0.904			0.938			0.067
Massive or Nested	11	12.52 (10.31, 13.34)		11	18.15 (17.78, 20.38)		26	3.63 (1.31, 18.96)	
Diffuse Infiltrative or Infiltralive	55	12.22 (11.28, 13.67)		55	18.47 (17.42, 20.07)		4	1.17 (0.45, 1.89)	

Note: CON, control; GC, gastric cancer. The higher ΔCt value indicates a lower expression while the higher 2^-ΔCt^ value means the higher expression level.

## Abbreviations

PGC: Pepsinogen C
GC: gastric cancer
PG: pepsinogen
SG: superficial gastritis
AG: atrophic gastritis
3'-UTR: 3'-Untranslated region
MRE: MiRNA response element
NC: Negative control
OS: Overall survival
