# Supplementary material for: Identification of PGC-related ncRNAs and their relationship with the clinicopathological features of Gastric Cancer
Source: J Cancer. 2021 May 27;12(14):4389–98. doi: 10.7150/jca.47787 (PMC8176405; doi:10.7150/jca.47787)
Supplement: Supplementary file 1 — Supplementary figure and tables. [file jcav12p4389s1.pdf]

**Supplementary Materials**

**Contents**

Figure S1：Fluorescent images of the cell transfection efficiency

Table S1: Primers of candidate circRNAs for qRT-PCR

Table S2: Sequence information of these 39 candidate miRNAs

Table S3: The screening of miRNAs by using transfecting mimics

Table S4: The screening of miRNAs by using miRNA overexpression plasmids

Table S5: Correlation between has-let-7c, hsa_circ_0001483 and hsa_circ_0001324 and environment factors

Table S6: Clinicopathological parameters of patients and univariate analysis of overall survival

Table S7: Univariate and multivariate cox proportional hazard analysis for the association of circRNAs and GC in tumor and normal tissues

**Figure Legend**

Figure S1: Fluorescent images of the cell transfection efficiency. A,B. Bright field and fluorescent images of AGS cells. C,D. Bright field and fluorescent images of HGC-27 cells. E,F. Bright field and fluorescent images of MKN-45 cells.

Figure S1

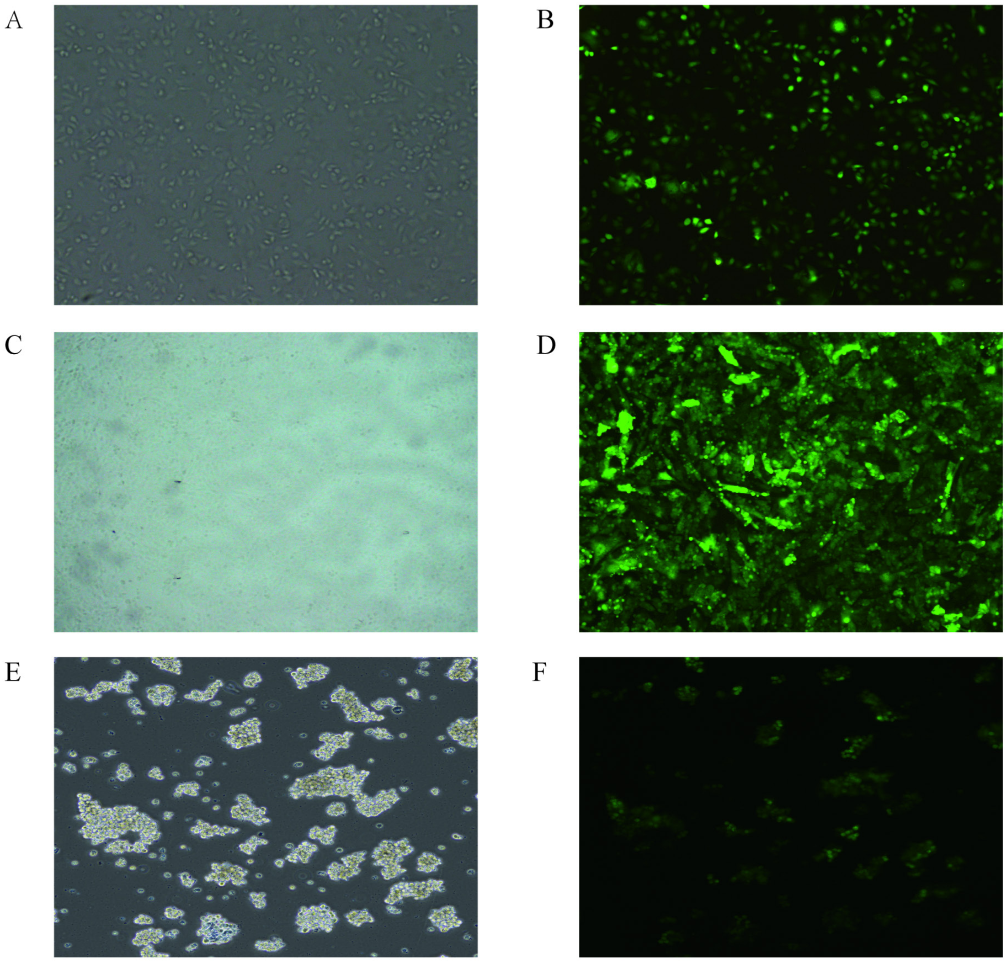

**Table S1. Primers of candidate circRNAs for qRT-PCR**

| Names | Primiers |
|---|---|
| hsa_circ_0001483F | AGCACGACAAGAAGGAGGTT |
| hsa_circ_0001483R | AGTGATACCCGTCTCTGCAA |
| hsa_circ_0001610F | ATGACTCCAACCCAGAACCG |
| hsa_circ_0001610R | CACGGTCAACATGGCGGTAT |
| hsa_circ_0001614F | ATCCCAGAAAGTTGCTGATAGGA |
| hsa_circ_0001614R | TTCCAAACCGGGGAAACAAAC |
| hsa_circ_0001685F | AGTGGAAGTGTCACCCTAAAAA |
| hsa_circ_0001685R | AGGTCTATTTCTTCTGCTCCAGT |
| hsa_circ_0012126F | TCCACCCTCAACCCATCTTCC |
| hsa_circ_0012126R | CGCAGACTCCACAGAAGAGGT |
| hsa_circ_0000365F | CTTCTTGGGAGACGGTCCAC |
| hsa_circ_0000365R | GCCACGGCGATAAGGAAAAT |
| hsa_circ_0000149F | AAGCCAAGAACGGTGAAAAGG |
| hsa_circ_0000149R | GCGAGCTCTTGACCTTGAAT |
| hsa_circ_0002557F | CCCCAACACCTTCAGGAAAAC |
| hsa_circ_0002557R | CGGCCTCTGCTCTATTTCTCC |
| hsa_circ_0000504F | GGCGAAGCGGATGGAATACA |
| hsa_circ_0000504R | CTTGTCGTGCCACAGTCGAT |
| hsa_circ_0001324F | TAGCTGATTGGTGGGCTGTT |
| hsa_circ_0001324R | TCGTTTTCCAACCCCTTCTCC |

| | |
|---|---|
| hsa_circ_1051F | GAAACCTGTGGAGCAGAAAGC |
| hsa_circ_1051R | GCGCAAAATCCCAGCCATAAG |
| hsa_circ_1355F | TATGGCTCCAGAAGGAAACGA |
| hsa_circ_1355R | GTATCTGATGACCGACGTCCC |

**Table S2. Sequence information of these 39 candidate miRNAs**

| Name | Sequence-forward | Sequence-reverse |
|---|---|---|
| hsa-mir-662 | 5' UCCCACGUUGUGGCCCAGCAG 3' | 5' GCUGGGCCACAACGUGGGAUU 3' |
| hsa-mir-198 | 5' GGUCCAGAGGGGAGAUAGGUUC 3' | 5' ACCUAUCUCCCCUCUGGACCUU 3' |
| hsa-let-7a | 5' UGAGGUAGUAGGUUGUAUAGUU 3' | 5' CUAUACAACCUACUACCUCAUU 3' |
| hsa-mir-320a | 5' AAAAGCUGGGUUGAGAGGGCGA 3' | 5' GCCCUCUCAACCCAGCUUUUUU 3' |
| hsa-mir-365 | 5' UAAUGCCCCUAAAAAUCCUUAU 3' | 5' AAGGAUUUUUAGGGGCAUUAUU 3' |
| hsa-mir-126-5p | 5' CAUUAUUACUUUUGGUACGCG 3' | 5' CGUACCAAAAGUAAUAAUGUU 3' |
| hsa-mir-520a-5p | 5' CUCCAGAGGGAAGUACUUUCU 3' | 5' AAAGUACUUCCCUCUGGAGUU 3' |
| hsa-mir-525-5p | 5' CUCCAGAGGGAUGCACUUUCU 3' | 5' AAAGUGCAUCCCUCUGGAGUU 3' |
| hsa-mir-939 | 5' UGGGGAGCUGAGGCUCUGGGGGUG 3' | 5' CCCCCAGAGCCUCAGCUCCCCAUU 3' |
| hsa-mir-185 | 5' UGGAGAGAAAGGCAGUUCCUGA 3' | 5' AGGAACUGCCUUUCUCUCCAUU 3' |

| | | |
|---|---|---|
| hsa-mir-98 | 5' UGAGGUAGUAAGUUGUAUUGUU 3' | 5' CAAUACAACUUACUACCUCAUU 3' |
| hsa-let-7b | 5' UGAGGUAGUAGGUUGUGUGGUU 3' | 5' CCACACAACCUACUACCUCAUU 3' |
| hsa-let-7c | 5' UGAGGUAGUAGGUUGUAUGGUU 3' | 5' CCAUACAACCUACUACCUCAUU 3' |
| hsa-let-7d | 5' AGAGGUAGUAGGUUGCAUAGUU 3' | 5' CUAUGCAACCUACUACCUCUUU 3' |
| hsa-mir-363-5p | 5' CGGGUGGAUCACGAUGCAAUUU 3' | 5' AUUGCAUCGUGAUCCACCCGUU 3' |
| hsa-mir-518f-5p | 5' CUCUAGAGGGAAGCACUUUCUC 3' | 5' GAAAGUGCUUCCCUCUAGAGUU 3' |
| hsa-let-7e | 5' UGAGGUAGGAGGUUGUAUAGUU 3' | 5' CUAUACAACCUCCUACCUCAUU 3' |
| hsa-let-7f | 5' UGAGGUAGUAGAUUGUAUAGUU 3' | 5' CUAUACAAUCUACUACCUCAUU 3' |
| hsa-let-7g | 5' UGAGGUAGUAGUUUGUACAGUU 3' | 5' CUGUACAAACUACUACCUCAUU 3' |
| hsa-let-7i | 5' UGAGGUAGUAGUUUGUGCUGUU 3' | 5' CAGCACAAACUACUACCUCAUU 3' |
| hsa-mir-10b-3p | 5' ACAGAUUCGAUUCUAGGGGAAU 3' | 5' UCCCCUAGAAUCGAAUCUGUUU 3' |
| hsa-mir-135b-3p | 5' AUGUAGGGCUAAAAGCCAUGGG 3' | 5' CAUGGCUUUUAGCCCUACAUUU 3' |
| hsa-mir-1827 | 5' UGAGGCAGUAGAUUGAAU 3' | 5' UCAAUCUACUGCCUCAUU 3' |

| | | |
|---|---|---|
| hsa-mir-335 | 5' UCAAGAGCAAUAACGAAAAAUGU 3' | 5' AUUUUUCGUUAUUGCUCUUGAUU 3' |
| hsa-mir-4283 | 5' UGGGGCUCAGCGAGUUU 3' | 5' ACUCGCUGAGCCCCAUU 3' |
| hsa-mir-4795-3p | 5' AUAUUAUUAGCCACUUCUGGAU 3' | 5' CCAGAAGUGGCUAAUAAUAUUU 3' |
| hsa-mir-493-3p | 5' UGAAGGUCUACUGUGUGCCAGG 3' | 5' UGGCACACAGUAGACCUUCAUU 3' |
| hsa-mir-518d-5p | 5' CUCUAGAGGGAAGCACUUUCUG 3' | 5' GAAAGUGCUUCCCUCUAGAGUU 3' |
| hsa-mir-518e-5p | 5' CUCUAGAGGGAAGCGCUUUCUG 3' | 5' GAAAGCGCUUCCCUCUAGAGUU 3' |
| hsa-mir-526a | 5' CUCUAGAGGGAAGCACUUUCUG 3' | 5' GAAAGUGCUUCCCUCUAGAGUU 3' |
| hsa-mir-526c | 5' CUCUAGAGGGAAGCGCUUUCUG 3' | 5' GAAAGCGCUUCCCUCUAGAGUU 3' |
| hsa-mir-532 | 5' CAUGCCUUGAGUGUAGGACCGU 3' | 5' GGUCCUACACUCAAGGCAUGUU 3' |
| hsa-mir-539 | 5' GGAGAAAUUAUCCUUGGUGUGU 3' | 5' ACACCAAGGAUAAUUUCUCCUU 3' |
| hsa-mir-583 | 5' CAAAGAGGAAGGUCCCAUUAC 3' | 5' AAUGGGACCUUCCUCUUUGUU 3' |
| hsa-mir-601 | 5' UGGUCUAGGAUUGUUGGAGGAG 3' | 5' CCUCCAACAAUCCUAGACCAUU 3' |
| hsa-mir-627 | 5' GUGAGUCUCUAAGAAAAGAGGA 3' | 5' CUCUUUUCUUAGAGACUCACUU 3' |

| | | |
|---|---|---|
| hsa-mir-759 | 5' GCAGAGUGCAAACAAUUUUGAC 3' | 5' CAAAAUUGUUUGCACUCUGCUU 3' |
| hsa-mir-875-3p | 5' CCUGGAAACACUGAGGUUGUG 3' | 5' CAACCUCAGUGUUUCCAGGUU 3' |
| hsa-mir-885-3p | 5' AGGCAGCGGGGUGUAGUGGAUA 3' | 5' UCCACUACACCCCGCUGCCUUU 3' |
| hsa-miR-16 (endogenous reference control) | 5' UAGCAGCACGUAAAUAUUGGCG 3' | 5' CCAAUAUUUACGUGCUGCUAUU 3' |

**Table S3. The screening of miRNAs by transfecting mimics**

| miRNAs | The average of luc/renilla | The standard deviation | The average of every miRNA/The average of no miRNA group |
|---|---|---|---|
| No RNA | 2.131834816 | 0.018534865 | 1.00 |

| | | | |
|---|---|---|---|
| hsa-miR-662 | 1.04341438 | 0.128299676 | 0.49 |
| hsa-miR-365 | 1.138878923 | 0.071279693 | 0.53 |
| hsa-let-7f | 1.663178636 | 0.051399805 | 0.78 |
| hsa-miR-98 | 1.676506245 | 0.082009321 | 0.79 |
| hsa-miR-525-5p | 1.696141786 | 0.117231313 | 0.80 |
| hsa-miR-520a-5p | 1.723876161 | 0.17534501 | 0.81 |
| hsa-let-7i | 1.803890037 | 0.242644251 | 0.85 |
| hsa-miR-126-5p | 1.819700798 | 0.222913827 | 0.85 |
| hsa-let-7c | 1.898091117 | 0.127006131 | 0.89 |
| hsa-let-7b | 1.949973736 | 0.19906586 | 0.91 |
| hsa-miR-939 | 1.978972968 | 0.112297242 | 0.93 |
| hsa-let-7a | 2.050810576 | 0.117018488 | 0.96 |
| hsa-miR-320a | 2.067392733 | 0.269203011 | 0.97 |

| | | | |
|---|---|---|---|
| hsa-let-7g | 2.101607236 | 0.049195764 | 0.99 |
| hsa-miR-518f-5p | 2.116382415 | 0.063327551 | 0.99 |
| hsa-let-7e | 2.167361007 | 0.178667206 | 1.02 |
| hsa-miR-198 | 2.287032242 | 0.115654225 | 1.07 |
| hsa-miR-363-5p | 2.408342415 | 0.112103244 | 1.13 |
| hsa-miR-185 | 2.424203146 | 0.308621272 | 1.14 |
| hsa-miR-4795-3p | 2.521273286 | 0.211477306 | 1.18 |
| hsa-miR-526a | 3.019809922 | 0.209309968 | 1.42 |
| hsa-miR-518e-5p | 3.110414738 | 0.131294206 | 1.46 |
| hsa-miR-885-3p | 3.114338933 | 0.243632844 | 1.46 |
| hsa-miR-518d-5p | 3.142133001 | 0.178387804 | 1.47 |
| hsa-miR-627 | 3.248331916 | 0.148712329 | 1.52 |
| hsa-miR-601 | 3.331457544 | 0.085947965 | 1.56 |

| | | | |
|---|---|---|---|
| hsa-miR-4283 | 3.377654344 | 0.052081429 | 1.58 |
| hsa-miR-1827 | 3.392767189 | 0.16056333 | 1.59 |
| hsa-miR-875-3p | 3.415138075 | 0.255779662 | 1.60 |
| hsa-miR-493-3p | 3.466340021 | 0.128688117 | 1.63 |
| hsa-miR-135b-3p | 3.469067325 | 0.102132528 | 1.63 |
| hsa-miR-10b-3p | 3.608660244 | 0.142712445 | 1.69 |
| hsa-miR-759 | 3.617386342 | 0.235793117 | 1.70 |
| hsa-miR-583 | 3.678820932 | 0.085209955 | 1.73 |
| hsa-miR-539 | 3.693944749 | 0.28324481 | 1.73 |
| hsa-miR-532 | 3.872661435 | 0.219379495 | 1.82 |
| hsa-let-7d | 3.912028155 | 0.164350754 | 1.84 |
| hsa-miR-526c | 4.033709788 | 0.046227977 | 1.89 |
| hsa-miR-335 | 4.530819415 | 0.166705744 | 2.13 |

| | | | |
|---|---|---|---|
| hsa-miR-16 | 3.03281007 | 0.17715557 | 1.42 |

**Table S4. The screening of miRNAs by using miRNA overexpression plasmids**

| miRNAs | The average of luc/renilla | The standard deviation | The average of every miRNA/The average of no miRNA group |
|---|---|---|---|
| No miRNA | 0.307449181 | 0.017530277 | 1.00 |
| hsa-miR-365 | 0.246686788 | 0.00267131 | 0.80 |
| hsa-miR-520a | 0.24710689 | 0.002081163 | 0.80 |
| hsa-let-7f | 0.254508583 | 0.006503693 | 0.83 |

| | | | |
|---|---|---|---|
| hsa-let-7c | 0.256151125 | 0.009799912 | 0.83 |
| hsa-miR-98 | 0.26191731 | 0.004995316 | 0.85 |
| hsa-miR-525 | 0.272758824 | 0.010348326 | 0.89 |
| hsa-miR-662 | 0.27506534 | 0.009631735 | 0.89 |
| hsa-miR-126 | 0.276722642 | 0.004809872 | 0.90 |
| hsa-let-7i | 0.282080068 | 0.0057359 | 0.92 |

**Table S5. Correlation between hsa-let-7c, hsa_circ_0001483 and hsa_circ_0001324 and environment factors**

| | N | Age | | Gender | | Smoking | | Alcohol Drinking | | Family History | | Peritumoral Inflammatory Cells | | Lymphatic metastasis | |
|---|---|---|---|---|---|---|---|---|---|---|---|---|---|---|---|
| GC | | r | *P* | r | *P* | r | *P* | r | *P* | r | *P* | r | *P* | r | *P* |
| hsa-let-7c | | 0.002 | 0.990 | 0.023 | 0.903 | 0.136 | 0.474 | 0.139 | 0.464 | 0.07 | 0.714 | 0.319 | 0.092 | 0.009 | 0.964 |

| | | | | | | | | | | | | | | | |
|---|---|---|---|---|---|---|---|---|---|---|---|---|---|---|---|
| hsa_circ_0001483 | 66 | -0.077 | 0.538 | -0.164 | 0.189 | 0.135 | 0.278 | 0.096 | 0.442 | 0.094 | 0.453 | 0.195 | 0.120 | **-0.249** | **0.044** |
| hsa_circ_0001324 | 66 | **-0.350** | **0.004** | 0.013 | 0.916 | 0.039 | 0.756 | 0.038 | 0.76 | 0.020 | 0.874 | 0.095 | 0.454 | -0.120 | 0.336 |

Note: The correlation analysis was performed by Spearman's correlation coefficient for the skewed ditribution data. GC, gastric cancer; r, Spearman's correlation coefficient.

**Table S6. Clinicopathological parameters of patients and univariate analysis of overall survival**

| Factors | | All GC n (%) | Death n (%) | MST[a] (M) | *P*-value |
|---|---|---|---|---|---|
| Cases | | 66 | 26 | | |
| Gender | | | | | 0.897 |
| | Male | 42(63.64) | 17(65.38) | 18.8[b] | |
| | Female | 24(36.36) | 9(24.62) | 18.8[b] | |

| | | | | | |
|---|---|---|---|---|---|
| Age | | | | | 0.116 |
| | ⩾60 | 38(57.58) | 18(69.23) | 17.6[b] | |
| | <60 | 28(42.42) | 8(30.77) | 20.5[b] | |
| Smoking | | | | | 0.658 |
| | Ever Smoker | 16(24.24) | 7(26.92) | 17.8[b] | |
| | Never Smoker | 50(75.76) | 19(73.08) | 19.2[b] | |
| Drinking | | | | | **0.034** |
| | Ever Drinker | 9(13.64) | 6(23.08) | 10.0 | |
| | Never Drinker | 57(86.36) | 20(76.92) | 19.5[b] | |
| Family History | | | | | 0.995 |
| | Yes | 15(22.73) | 6(23.08) | 19.0[b] | |
| | No | 51(77.27) | 20(76.92) | 18.8[b] | |
| Location | | | | | **0.029** |

| | | | | | |
|---|---|---|---|---|---|
| | Body | 20(30.77) | 11(42.31) | 18.0 | |
| | Angle | 11(16.92) | 1(3.85) | 23.1[b] | |
| | Antrum | 23(35.38) | 7(26.92) | 20.0[b] | |
| | Entire | 11(16.92) | 7(26.92) | 10.0 | |
| Macroscopic Type | | | | | 0.087 |
| | Protruded Type | 3(4.55) | 1(3.85) | 22.0[b] | |
| | Ulcerative Type | 13(19.70) | 2(7.69) | 21.6[b] | |
| | Ulcerative Infiltrative Type | 32(48.48) | 12(46.15) | 19.2[b] | |
| | Diffuse Infiltrative Type | 18(27.27) | 11(42.31) | 10.0 | |
| Histological Type | | | | | 0.159 |
| | Papillary Adenocarcinoma (I) | 0 | 0 | NA | |
| | Well Differentiated Type (II) | 1(1.56) | 0 | NA | |
| | Moderately Differentiated Type (III) | 8(12.50) | 2(7.69) | NA | |

| | | | | | |
|---|---|---|---|---|---|
| | Poorly Differentiated Type (IV) | 39(60.94) | 19(73.08) | NA | |
| | Mucinous Adenocarcinoma (V) | 6(9.38) | 0 | NA | |
| | Signet-ring Cell Cinoma (VI) | 10(15.63) | 5(19.23) | NA | |
| Lauren Classification | | | | | |
| | Intestinal | 55(85.94) | 24(92.31) | 23.2[b] | 0.183 |
| | Diffuse | 9(14.06) | 2(7.69) | 17.9[b] | |
| TNM stage | | | | | **0.001** |
| | I | 7(10.61) | 0 | NA | |
| | II | 23(34.85) | 5(19.23) | NA | |
| | III | 36(54.55) | 21(90.77) | NA | |
| | IV | 0 | 0 | NA | |
| | | | | | **<0.001** |
| | Ⅰ+Ⅱ | 30(45.45) | 5(19.23) | 22.4[b] | |

| | | | | | |
|---|---|---|---|---|---|
| | III+IV | 36(56.06) | 21(80.77) | 13.0 | |
| Peritumoral Inflammatory Cells | | | | | 0.211 |
| | + | 18(27.69) | 9(34.62) | 18.0 | |
| | ++ | 26(40.00) | 7(26.92) | 20.6[b] | |
| | +++ | 21(32.31) | 10(38.46) | 18.3[b] | |
| Lymphovascular invasion | | | | | 0.067 |
| | + | 42(63.64) | 20(76.92) | 17.6[b] | |
| | - | 24(36.36) | 6(23.08) | 21.0[b] | |
| Ganglion Invasion | | | | | 0.071 |
| | + | 53(81.54) | 24(92.31) | 17.8[b] | |
| | - | 12(32.31) | 2(7.69) | 22.8[b] | |
| Lymphatic metastasis | | | | | **0.001** |
| | + | 45(68.18) | 24(92.31) | 18.0 | |

| | | | | | |
|---|---|---|---|---|---|
| | - | 21(31.82) | 2(7.69) | 23.4[b] | |
| Depth of Invasion | | | | | |
| | Mucous Layer (pT1) | 2(3.03) | 0 | NA | 0.088 |
| | Submucosal Layer (pT2) | 5(7.58) | 0 | NA | |
| | Muscular Layer (pT3) | 7(10.61) | 1(3.85) | NA | |
| | Subserosa Layer (pT4) | 1(1.52) | 0 | NA | |
| | Serosal Layer or Invasion Adjacent Organs(pT5) | 51(77.27) | 25(96.15) | NA | |
| | | | | | **0.018** |
| | pT1+pT2 | 7(10.61) | 0 | NA | |
| | pT3+pT4 | 8(12.12) | 1(3.85) | NA | |
| | pT5 | 51(77.27) | 25(96.15) | NA | |
| Growth Pattern | | | | | 0.107 |
| | Massive or Nested | 11(16.67) | 2(7.69) | 23.0[b] | |

| | | | |
|---|---|---|---|
| Diffuse Infiltrative or Infiltralive | 55(83.33) | 24(92.31) | 18.0[b] |

Note: a, MST, median survival time (months); b, mean survival time was provided when MST could not be calculated; GC, gastric cancer; NA, not avaliable.

**Table S7. Univariate and multivariate cox proportional hazard analysis for the association of circRNAs and GC in tumor and normal tissues**

| | | All GC | Death | MST[a] | Univariate | | Multivariate[c] | |
|---|---|---|---|---|---|---|---|---|
| Variables | | n | n (%) | (M) | P-value | Hazard ratio (95% CI) | P-value | Hazard ratio (95% CI) |
| has_circ_1324 | Low | 33 | 15(57.69) | 18.0[b] | 0.328 | 1(ref) | 0.070 | 1(ref) |
| | High | 33 | 11(42.30) | 19.6[b] | | 0.67(0.31-1.48) | | 0.48(0.21-1.06) |
| has_circ_1483 | Low | 33 | 10(38.46) | 20.0[b] | 0.164 | 1(ref) | | 1(ref) |
| | High | 33 | 16(61.54) | 17.6[b] | | 0.56(0.25-1.23) | 0.081 | 0.42(0.16-1.11) |

Note: GC, gastric cancer; HR, hazard rate; CI, confidence interval; a, MST, median survival time (months); b, mean survival time was provided when MST could not be

calculated; c, Multivariate survival analysis was carried out by adding the clinicopathological parameters with $P<0.05$. NA: not available.
